# Fish Oil And/Or Probiotics Intervention in Overweight/Obese Pregnant Women and Overweight Risk in 24-Month-Old Children

**DOI:** 10.1097/MPG.0000000000003659

**Published:** 2022-11-23

**Authors:** Lotta Saros, Tero Vahlberg, Ella Koivuniemi, Noora Houttu, Harri Niinikoski, Kristiina Tertti, Kirsi Laitinen

**Affiliations:** From the * Institute of Biomedicine, Integrative Physiology and Pharmacology, University of Turku, Turku, Finland; the † Institute of Clinical Medicine and Biostatistics, University of Turku, Turku, Finland; the ‡ Department of Pediatrics and Adolescent Medicine, Turku University Hospital, Turku, Finland; the § Department of Obstetrics and Gynecology, Turku University Hospital and University of Turku, Turku, Finland; ‖ Functional Foods Forum, University of Turku, Turku, Finland.

**Keywords:** children, fish oil, growth, overweight, probiotics

## Abstract

To evaluate whether a fish oil and/or probiotics intervention in pregnant women with overweight or obesity would influence the tendency of their 24-month-old children to become overweight and alter their body fat percentage.

**Methods:**

Women (n = 439) were double-blindly randomized into 4 intervention groups: fish oil+placebo, probiotics+placebo, probiotics+fish oil, and placebo+placebo (fish oil: 1.9 g docosahexaenoic acid and 0.22 g eicosapentaenoic acid, probiotics: *Lacticaseibacillus rhamnosus* HN001 and *Bifidobacterium animalis* ssp. *lactis* 420, 10^10^ colony-forming units each). The intervention lasted from early pregnancy until 6 months postpartum. Children’s (n = 330) growth data (height, weight, head circumference), a secondary outcome of the trial, were evaluated at birth, 3, 6, 12, and 24 months of age and compared to Finnish growth charts. Body fat percentage was measured with air displacement plethysmography (24 months). Logistic regression and general linear models were used to analyze the data.

**Results:**

Probiotics+placebo [weight-for-height% adj. Odds ratio (OR) = 0.36, 95% confidence interval (CI) = 0.14–0.95] and probiotics+fish oil [weight-for-age standard deviation score (SD-score) adj. OR = 0.22, 95% CI = 0.07–0.71] associated with lower overweight odds in 24-month-old children compared to placebo+placebo. Results remained essentially the same, when probiotics’ main effect (combined probiotics+placebo and probiotics+fish oil) was estimated; that is, lower overweight odds (weight-for-height% adj. OR = 0.48, 95% CI = 0.25–0.95 and weight-for-age SD-score adj. OR = 0.42, 95% CI = 0.20–0.88) compared to non-probiotics. No fish oil main effect (combined fish oil+placebo and probiotics+fish oil) was seen. The intervention did not influence body fat percentage.

**Conclusions:**

The administration of probiotics solely and in combination with fish oil during pregnancy to women with overweight or obesity lowered the overweight odds of their 24-month-old children.

**What Is Known**

- Children of mothers with overweight or obesity are at an increased risk for overweight in later life.
- Preliminary evidence indicates that probiotics and fish oil have beneficial effects on child growth, but their combined effects is not known.

**What Is New**

- Administration of probiotics alone and in combination with fish oil during pregnancy lowered the risk of child being overweight at the age of 24 months.
- The intervention with probiotics and fish oil may benefit the growth of children of mothers with overweight or obesity.

Pregnancy is a continuum of various physiological adjustments, including those occurring in glucose and insulin metabolism to ensure the proper growth of the fetus (1). Maternal adiposity poses an additional burden on the metabolism including insulin resistance and low-grade inflammation (2). Subsequently, the fetus is at an increased risk for higher birthweight (3) and even obesity later in life (4), probably through early programming mechanisms (5). Modification of early life circumstances, for example, by fish oil and/or probiotics administration, could be a feasible way to support child’s growth.

The evidence on the effects of fish oil, particularly long-chain polyunsaturated fatty acid (LC-PUFA), and probiotics on child’s growth is limited, but some promising reports do exist. Previously fish oil supplementation during pregnancy has been associated with a higher birth weight, height, and greater head circumference (6–8), and with a higher body lean mass at birth, but not with body fat percentage or fat mass (8). Further, LC-PUFA intake during pregnancy has lowered obesity risk of 3-year-old children (9). Probiotics are shown to exert beneficial effects on the adult’s weight (10–12), but their impact on child’s weight has been less studied. In 1 trial, probiotics administration during pregnancy is associated with lower excessive weight-gain in 4-year-old children (13). However, not all studies have found associations between these supplements and child’s growth, weight, or body composition (14–16). Fish oil and probiotics potentially have co-effects on maternal metabolism (17), but this view has not been yet studied with regards to child’s growth. We hypothesized that fish oil and/or probiotics supplementation during pregnancy is beneficial for the child’s growth through their effects in regulating low-grade inflammation and insulin metabolism (18–21). Our objective was to investigate whether a fish oil and/or probiotics intervention of women with overweight/obesity influence their 24-month-old child’s overweight and fat percentage. Furthermore, the intervention effects on height, weight, and head circumference from 3 to 24 months were evaluated.

## METHODS

### Study Design and Participants

**FIGURE 1.**
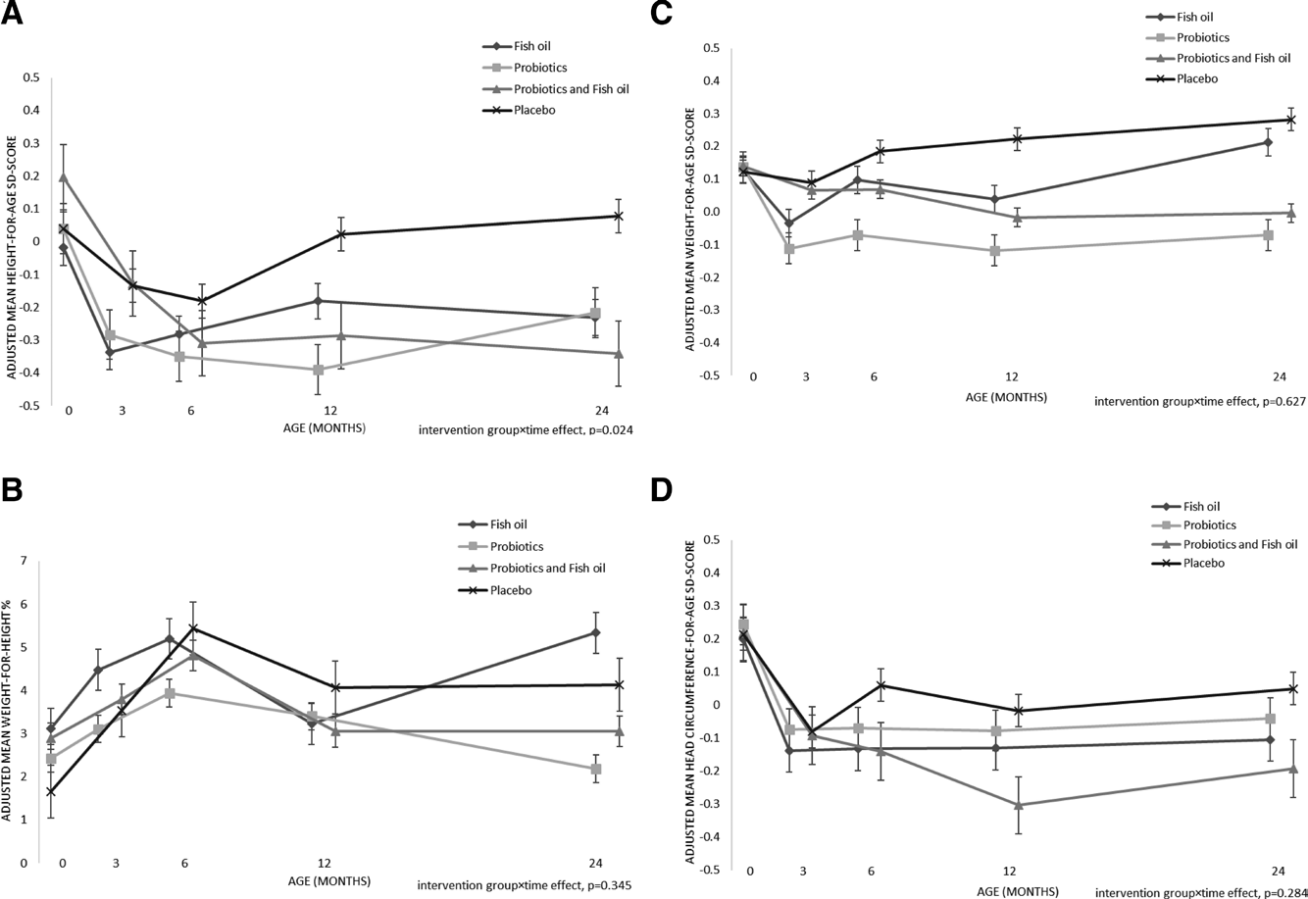
Interaction between the intervention group and time after birth on the children’s growth markers over the study period. Figure shows the adjusted mean (standard error) for (A) height-for-age SD-score, (B) weight-for-height%, (C) weight-for-age SD-score, and (D) head circumference-for-age SD-score derived from analysis of covariance for repeated measurements. Analyses were adjusted for maternal smoking status before pregnancy and for the child’s birth weight. Significant interaction between intervention group and time in the height-for-age SD-score; in the subsequent pairwise interaction effect comparisons, the difference was evident between the probiotics+fish oil group and placebo+placebo group (group × time effect, *P* = 0.02). SD = standard deviation.

In this sub-study of a double-blind, placebo-controlled randomized trial, we investigated whether the administration of fish oil and/or probiotics supplements to pregnant women with overweight/obesity could affect overweight and fat percentage of their 24-month-old children. Secondly, we investigated the intervention’s effects on the child’s height, weight, and head circumference. The trial design has been described previously (22) (ClinicalTrials.gov Identifier: NCT01922791). Briefly, the inclusion criteria were early pregnancy (<18 gestational weeks), pre-pregnancy body mass index (BMI) ≥ 25 kg/m^2^, and no presence of chronic diseases. A total of 439 women were recruited in Southwest Finland (October 2013–July 2017). For this study, we included those children in whom we had growth data from at least 1 measuring point over the 24-months’ study period, the total sample being 330 child-mother-dyads (Figure 1, Supplemental Digital Content 1, http://links.lww.com/MPG/C990). Two study visits were arranged during pregnancy and 4 after delivery (3, 6, 12, and 24 months). The women filled in 3-day food diaries in early pregnancy, from which dietary patterns, a healthier and an unhealthier (data not shown), were identified as described previously (23). The study was carried out according to the guidelines laid down in the Declaration of Helsinki. The Ethics Committee of the Hospital District of Southwest Finland approved the study protocol. Each participant provided written informed consent before participation.

### Fish Oil And/Or Probiotics Supplementation

At the baseline (13.9 ± 2.1 gestational weeks), the women were double-blindly randomized into 4 parallel intervention groups (fish oil+placebo, probiotics+placebo, probiotics+fish oil, placebo+placebo), and the intervention lasted until 6 months post-partum. Fish oil capsules (Croda Europe Ltd., Leek, UK) contained 2.4 g n-3 fatty acids: 1.9 g docosahexaenoic acid (DHA, 22:6-n-3), 0.22 g eicosapentaenoic acid (EPA, 20:5-n-3), and other n-3 fatty acids, including docosapentaenoic acid. Placebo capsules contained medium-chain fatty acids, such as capric acid. The probiotic capsules contained 10^10^ colony-forming units of *Lacticaseibacillus rhamnosus* HN001 (formerly *Lactobacillus rhamnosus* HN001) (ATCC SD5675; DuPont, Niebüll, Germany) and *Bifidobacterium animalis* ssp*. lactis* 420 (DSM 22089; DuPont). Placebo capsules consisted of microcrystalline cellulose. The size, shape, and color of placebo capsules were identical to the intervention capsules. The women were instructed to take 2 fish oil capsules and 1 probiotic capsule daily. Allocation into intervention groups was conducted according to women’s parity and gestational diabetes mellitus (GDM) history (primipara, multipara, multipara + previous GDM). The stratified randomization was performed with 4 random permuted blocks, and a statistician (not involved in recruitment or study execution) generated randomization lists of the 3 blocks. Altogether, 88.4% women reported good compliance with the intervention (17). These supplements were selected based on the previous scientific knowledge. *L rhamnosus* HN001 is a well characterized probiotic (24). *B animalis* ssp. *lactis* 420, a novel probiotic, and fish oil, particularly LC-PUFA, influence beneficially insulin metabolism and inflammation (18,19,25,26). Additionally, LC-PUFA is needed in child’s growth and development (27).

### Childs’s Anthropometrics and Body Composition

Children’s growth data were obtained from child welfare clinic cards during the study visits. Weight-for-height%, weight-, height-, head circumference-, and BMI-for-age standard deviation (SD)-scores were calculated according to Finnish growth references (28,29). Appropriate growth references (30) were used for preterm children (n = 24). The BMI-for-age SD-score (n = 149) was calculated for children whose age was ≥1.995 years. A weight-for-age SD-score was categorized into normal/underweight, overweight, and obese (≤1 SD, >1–2 SD, and >2 SD, respectively), weight-for-height% into normal/underweight, overweight, and obese (<+10%, +10–20%, and >+20%, respectively), and BMI-for-age SD-score into normal/underweight, overweight, and obese (girls: <1.1629 SD, 1.1629–1.1629 SD, and ≥2.1065 SD, and boys:<0.7784 SD, 0.7784–0.7784 SD, and ≥1.7016 SD) (29). Since the numbers of children with underweight were small (n = 9/250, 3.6%) they were combined with the children with normal weight (normal weight + underweight). Correspondingly, the children with obesity (n = 14/250, 5.6%) were combined with the children with overweight (overweight + obese). These groups are hereafter referred to as normal weight and overweight.

The body composition of children was measured using air displacement plethysmography (the Bod Pod-system, software version 5.4.0, COSMED, Inc., Concord, CA) according to the manufacturer’s instructions, applying the pediatric option in the Bod Pod-system. Children were measured wearing a tight cap and underwear/swimming trunks without a diaper. They did not have restrictions regarding eating or drinking. The density model devised by Fomonet al (31) was used to calculate body fat percentage (n = 73).

### Statistical Analysis

The normality of the data was evaluated visually using histograms. Normally distributed variables are described as mean ± SD, those not normally distributed as median (interquartile range), and categorical variables as frequency (%). One-way ANOVA and independent samples *t* test were used to compare normally distributed data, otherwise Kruskal-Wallis or Mann-Whitney *U*-test were used. Chi-squared or Fisher exact test was used for categorical data. The differences in the child’s overweight odds were evaluated using binary logistic regression models. Additionally, binary logistic regression models were used to analyze the main effects of fish oil and probiotics and a fish oil × probiotics interaction effect on the child’s overweight odds. General linear models were used to compare mean growth in height, weight, and head circumference between the groups. The impact of fish oil and probiotics on child’s growth measures was analyzed by general linear models with the main effects for fish oil and probiotics as well as a fish oil × probiotics interaction effect. Analyses of covariance for repeated measurements were used to evaluate the difference in the child’s growth between the groups over the 24-month study period. Fat percentage was not normally distributed and was natural log transformed in the statistical analyses. Analysis were adjusted for maternal prepregnancy smoking status as it differed between the groups at baseline, child’s age at the measurement (weight-for-height%), and birthweight as it is known to influence child’s growth and was associated with the outcomes. A 2-tailed *P* value < 0.05 was considered significant. The analyses were performed with IBM SPSS statistics version 27.0 for Windows (IBM SPSS Inc., Chicago, IL).

## Results

### Clinical Characteristics of Mothers and Children

The clinical characteristics of the mothers and their children are presented in Table 1. The women were generally highly educated, and every second woman was primipara. The majority of the women had overweight (60.6%) and the rest had obesity. Considering the intervention groups, the only difference was evident in the women’s prepregnancy smoking status; the proportion was highest in the placebo+placebo group. No differences in children’s characteristics were seen between the groups (Table 1). The majority of the children had normal weight (81.6%) and the rest had overweight (girls = 17.1%, boys = 19.7%, all = 18.4%), when evaluated by weight-for-height%, at 24 months of age (Table 2).

**TABLE 1.** Clinical characteristics of all mothers and children and according to the intervention groups

Characteristics	n	All	Fish oil + placebo	Probiotics + placebo	Probiotics + fish oil	Placebo + placebo	*P* value*
Mother baseline							
Age, y†	82/81/82/85	30.7 ± 4.5	30.5 ± 4.8	30.9 ± 4.3	31.1 ± 4.7	30.5 ± 4.1	0.698
College or university education‡	82/79/82/85	211 (63.9)	56 (68.3)	54 (68.4)	50 (61.0)	51 (60.0)	0.540
Primiparity‡	82/81/82/85	161 (48.8)	42 (51.2)	39 (48.1)	40 (48.8)	40 (47.1)	0.958
Smoked before pregnancy‡	82/79/81/85	57 (17.3)	8 (9.8)	20 (25.0)	6 (7.3)	23 (27.1)	<0.001
Pre-pregnancy BMI, kg/m^2^§	82/81/82/85	28.7 (26.5; 31.8)	29.4 (27.1; 32.7)	28.4 (26.5; 31.0)	28.3 (26.1; 31.9)	29.2 (26.5; 31.8)	0.267
Overweight‡	82/81/82/85	200 (60.6)	44 (53.7)	53 (65.4)	52 (63.4)	51 (60.0)	0.435
Obese‡		130 (39.4)	38 (46.3)	28 (34.6)	30 (36.6)	34 (40.0)	
Gestational weeks†	82/81/82/85	13.9 ± 2.1	13.8 ± 2.3	13.8 ± 2.1	14.1 ± 1.9	13.9 ± 2.0	0.826
Blood pressure, mmHg†							
Systolic	82/80/82/84	118 ± 10	116 ± 11	118 ± 11	116 ± 9.4	119 ± 9.4	0.345
Diastolic	82/80/82/84	77 ± 8.3	77 ± 9.1	76 ± 9.1	76 ± 7.3	77 ± 7.4	0.162
Mother pregnancy							
GDM diagnosis in current pregnancy‡	81/81/78/83	93 (28.9)	27 (33.3)	24 (29.6)	22 (28.2)	21 (25.3)	0.723
Smoked during pregnancy‡	82/80/82/85	13 (3.9)	0 (0)	4 (5.1)	3 (3.7)	6 (7.1)	0.077
Gestational weeks at delivery§	82/81/82/85	39.7 (39.0; 40.6)	40.0 (39.3; 40.5)	40.1 (39.0; 40.7)	39.6 (38.9; 40.7)	39.7 (38.6; 40.5)	0.564
Unassisted vaginal delivery‡	82/81/82/85	239 (72.4)	57 (69.5)	62 (76.5)	61 (74.4)	59 (69.4)	0.664
Healthier dietary pattern, early pregnancy‡	79/79/81/83	164 (50.9)	36 (45.6)	40 (50.6)	38 (46.9)	50 (60.2)	0.230
Child							
Gender, girl‡	82/81/82/85	165 (50.0)	41 (50.0)	40 (49.4)	44 (53.7)	40 (47.1)	0.862
Born preterm‡	82/81/82/85	19 (5.8)	4 (4.9)	4 (4.9)	8 (9.8)	3 (3.5)	0.386
SGA‡	82/81/82/85	9 (2.7)	0 (0)	2 (2.5)	3 (3.7)	4 (4.7)	0.250
LGA‡	82/81/82/85	16 (4.8)	3 (3.7)	5 (6.2)	4 (4.9)	4 (4.7)	0.895
Birth height	80/80/79/85	50.6 ± 2.31†	51.0 (50.0; 52.0)‡	51.0 (49.1; 52.0)‡	51.0 (49.0; 52.0)‡	50.5 (49.0; 52.0)‡	0.970
Birth weight†	82/81/82/85	3612 ± 545	3620 ± 537	3636 ± 547	3581 ± 589	3612 ± 513	0.933
Birth head circumference	80/80/78/85	35.2 ± 1.54†	35.0 (34.5; 36.0)‡	35.5 (34.0; 36.5)‡	35.0 (34.0; 36.0)‡	35.0 (34.0; 36.0)‡	0.833
Breast feeding, mo†	75/64/66/70	11.0 ± 6.71	10.7 ± 6.78	11.0 ± 7.25	11.8 ± 6.33	10.4 ± 6.56	0.684

Early pregnancy 13.9 ± 2.1 gestational weeks.

BMI = body mass index; GDM = gestational diabetes mellitus; LGA = large for gestational age; SD = standard deviation; SGA = small for gestational age.

* One-way ANOVA for normally distributed variables, otherwise Kruskal-Wallis H. Chi-squared test or Fisher exact test for categorical variables.

† Data are presented as mean ± SD.

‡ Data are presented as frequency (%).

§ Data are presented as median (interquartile range).

**TABLE 2.** Association between the intervention of fish oil and/or probiotics and children’s overweight risk at the age of 24 months

Growth measures	All	Fish oil + placebo	Adjusted *P* *	Probiotics + placebo	Adjusted *P* *	Probiotics + fish oil	Adjusted *P* *	Placebo + placebo
	n (%)	n (%)		n (%)		n (%)		n (%)
Weight-for-height%	n = 250							
Normal + Underweight	204 (81.6)	50 (80.6)		54 (87.1)		54 (85.7)		46 (73.0)
Overweight + Obese	46 (18.4)	12 (19.4)		8 (12.9)		9 (14.3)		17 (26.9)
Adjusted OR (95% CI)		0.77 (0.32–1.82)	0.546	0.36 (0.14–0.95)	0.038	0.50 (0.20–1.26)	0.142	1
Weigh-for-age SD-score	n = 250							
Normal + Underweight	209 (83.6)	52 (83.9)		52 (83.9)		59 (93.7)		46 (73.0)
Overweight + Obese	41 (16.4)	10 (16.1)		10 (16.1)		4 (6.4)		17 (26.9)
Adjusted OR (95% CI)		0.62 (0.25–1.55)	0.307	0.47 (0.19–1.18)	0.107	0.22 (0.07–0.71)	0.011	1
BMI-for-age SD-score	n = 149							
Normal + Underweight	113 (76.4)	27 (77.1)		32 (84.2)		27 (73.0)		27 (69.2)
Overweight + Obese	36 (24.2)	8 (22.8)		6 (15.8)		10 (27.0)		12 (30.8)
Adjusted OR (95% CI)		0.67 (0.23–1.97)	0.463	0.36 (0.11–1.13)	0.079	0.81 (0.29–2.30)	0.694	1

Data are presented as frequency (%). Weight-for-age SDS: normal + underweight ≤ 1, overweight + obese > 1 SD-score. Weight-for-height%: normal + underweight < 10, overweight + obese ≥ 10%. BMI-for-age SD-score: girls: overweight + obesity ≥ 1.1629, and boys: overweight + obesity ≥ 0.7784 SD-score.

BMI = body mass index; CI = confidence interval; OR = odds ratio; SD-score = standard deviation score.

* Binary logistic regression model for overweight with placebo group as the reference category. Adjusted for maternal smoking status before pregnancy, child’s birth weight, and child’s age at the measurement (weight-for-height%).

### Impact of the Intervention on Children’s Overweight Status at 24 Months of Age

We observed that maternal consumption of (1) probiotics+placebo and (2) probiotics+fish oil were associated with lower odds of child being overweight (1 = weight-for-height%, 2 = weight-for-age SD-score as outcomes) when compared to the placebo+placebo in the adjusted models (Table 2). We also investigated the main effects of fish oil (combined fish oil+placebo-group and probiotics+fish oil-group) and probiotics (combined probiotics+placebo-group and probiotics+fish oil-group) on the child’s overweight after checking that the interaction effect between fish oil and probiotics was not significant (Table 1, Supplemental Digital Content 2, http://links.lww.com/MPG/C991). We found that probiotics consumption was associated with lower overweight odds of child, using weight-for-height% and weight-for-age SD-score as outcomes, as compared to non-probiotics in the adjusted models (Table 1, Supplemental Digital Content 2, http://links.lww.com/MPG/C991). Fish oil administration did not influence the child’s overweight odds.

The children’s adiposity was investigated in more detail by measuring their body fat percentage at 24 months of age but no differences were detected between the intervention groups (Table 3). Furthermore, in the evaluation of the main effects, neither fish oil nor probiotics influenced the body fat percentage (Table 2, Supplemental Digital Content 3, http://links.lww.com/MPG/C997).

**TABLE 3.** Growth measurements of all children and when subdivided into the four different intervention groups

Growth measures	All mean ± SD	n	Fish oil + placebo adjusted mean (SE)	Probiotics + placebo adjusted mean (SE)	Probiotics + fish oil adjusted mean (SE)	Placebo + placebo adjusted mean (SE)	Adjusted *P*‡
3 months							
Age at measurement, y *	0.25 (0.25; 0.26)		0.25 (0.24; 0.26)	0.25 (0.25; 0.26)	0.25 (0.25; 0.26)	0.25 (0.25; 0.26)	
Height SD-score	−0.20 ± 1.11	82/81/81/83	−0.29 (0.12)	−0.25 (0.11)	−0.12 (0.12)	−0.12 (0.11)	0.548
Weight-for-height%	3.14 ± 8.40	82/81/81/83	4.43 (1.06)	3.21 (0.99)	3.64 (1.08)	3.42 (0.97)	0.814
Weight-for-age SD-score	−0.02 ± 0.97	82/81/81/83	0.03 (0.11)	−0.06 (0.10)	0.06 (0.11)	0.09 (0.10)	0.723
Head circumference-for-age SD-score	−0.07 ± 1.10	81/73/80/82	−0.02 (0.13)	−0.01 (0.12)	0.01 (0.13)	−0.03 (0.12)	0.994
6 months							
Age at measurement, y *	0.50 (0.49; 0.51)		0.50 (0.49; 0.51)	0.50 (0.50; 0.51)	0.50 (0.49; 0.51)	0.51 (0.50; 0.52)	
Height SD-score	−0.26 ± 1.11	79/69/78/74	−0.14 (0.13)	−0.24 (0.13)	−0.19 (0.13)	−0.13 (0.12)	0.907
Weight-for-height%	4.34 ± 8.47	79/69/78/74	5.26 (1.10)	4.21 (1.09)	4.64 (1.12)	5.38 (1.05)	0.827
Weight-for-age SD-score	0.07 ± 0.95	79/68/78/74	0.23 (0.12)	0.05 (0.12)	0.14 (0.19)	0.22 (0.11)	0.616
Head circumference-for-age SD-score	−0.03 ± 1.08	78/68/78/71	0.01 (0.13)	−0.02 (0.13)	0.02 (0.13)	0.17 (0.13)	0.675
12 months							
Age at measurement, y *	1.00 (0.99; 1.01)		1.00 (0.99; 1.01)	1.01 (0.99; 1.01)	1.00 (0.99; 1.02)	1.00 (0.99; 1.01)	
Height SD-score	−0.20 ± 1.08	74/66/71/71	−0.07 (0.14)	−0.22 (0.13)	−0.14 (0.14)	0.11 (0.13)	0.257
Weight-for-height%	2.76 ± 8.30	74/66/71/71	4.41 (1.09)	4.31 (1.05)	3.53 (1.11)	4.84 (1.02)	0.815
Weight-for-age SD-score	0.01 ± 0.98	74/66/71/71	0.23 (0.12)	0.12 (0.12)	0.11 (0.13)	0.35 (0.12)	0.357
Head circumference-for-age SD-score	−0.10 ± 1.09	72/63/70/71	−0.05 (0.14)	0.07 (0.14)	−0.20 (0.15)	0.13 (0.13)	0.276
24 months							
Age at measurement, y *	2.00 (1.98; 2.02)		2.00 (1.98; 2.02)	2.00 (1.98; 2.02)	2.00 (1.97; 2.02)	2.00 (1.98; 2.02)	
Height SD-score	−0.17 ± 1.06	62/62/63/63	−0.16 (0.15)	−0.11 (0.13)	−0.26 (0.15)	0.16 (0.13)	0.114
Weight-for-height%	2.91 ± 8.52	62/62/63/63	6.29 (1.19)	2.46 (1.09)	3.40 (1.19)	4.34 (1.09)	0.067
Weight-for-age SD-score	0.06 ± 0.99	62/62/63/63	0.33 (0.13)	0.06 (0.12)	0.05 (0.13)	0.36 (0.12)	0.112
Head circumference-for-age SD-score	−0.06 ± 1.06	60/56/62/58	−0.13 (0.15)	0.04 (0.14)	−0.17 (0.15)	0.18 (0.14)	0.243
BMI-for-age SD-score	0.28 ± 1.05	35/38/37/39	0.55 (0.19)	0.21 (0.18)	0.34 (0.19)	0.55 (0.18)	0.402
Fat mass (%)	24.6 ± 8.87	18/16/18/21	23.8 (19.7; 28.8)†	23.5 (19.2; 28.8)†	21.1 (17.3; 25.5)†	21.4 (17.9; 25.5)†	0.680

Data are presented as mean ± SD and adjusted mean (SE). Fat percentage ln transformed for the analysis due to skewed distribution.

BMI = body mass index; SD = standard deviation; SE = standard error.

* Data are presented as median (interquartile range).

† Data are presented as adjusted geometric mean (95% CI).

‡ General linear model adjusted for maternal smoking status before pregnancy, children’s birth weight, and age at the measurement (weight-for-height%).

### Impact of the Intervention on Children’s Height, Weight, and Head Circumference During the First 24 Months of Age

The mean growth of the children was within the normal reference range over the study period (Table 3). No differences in the growth markers between the intervention groups were seen at any time point (Table 3). We further investigated the main effects of fish oil (combined fish oil+placebo-group and probiotics+fish oil-group) and probiotics (combined probiotics+placebo-group and probiotics+fish oil-group) on the child’s growth markers as the interaction effect between fish oil and probiotics was not significant (Table 2, Supplemental Digital Content 3, http://links.lww.com/MPG/C997). After adjusting for confounders, it was found that maternal probiotics consumption was associated with lower weight-for-height% and weight-for-age SD-score of 24-month-old children (Table 2, Supplemental Digital Content 3, http://links.lww.com/MPG/C997). Fish oil consumption did not influence these growth markers.

We found that the intervention group and time had an interaction effect on the child’s height-for-age SD-score (intervention group × time effect, *P* = 0.02), but not on the weight or head circumference variables (Fig. 1). Specifically, the mean height-for-age SD-score decreased more in the probiotics+fish oil-group than the placebo+placebo-group (intervention group × time effect, *P* = 0.02) over the 24-months’ period (Fig. 1).

## DISCUSSION

We demonstrated that maternal consumption of *L rhamnosus* HN001 and *B animalis* ssp. *lactis* 420 solely and in combination with fish oil from early pregnancy onwards associated with lower overweight odds of their 24-month-old children. However, no intervention effect on the body fat percentage was seen. Although the mean growth was within the normal reference range, the combination of probiotics and fish oil associated with a lower height-for-age SD-score of the children when compared to placebo-group during the 24-months’ study period.

Our findings suggest that maternal probiotics consumption (10^10^ colony-forming units of *L rhamnosus* HN001 and *B animalis* ssp*. lactis* 420) lowered the overweight odds and weight of their children. The same result was seen with the combination of probiotics and fish oil. Our findings are in line with a previous Finnish study (n = 159) in which a probiotics supplementation (1 × 10^10^ colony-forming units of *L rhamnosus* GG) during pregnancy and for 6 months postpartum (to the breastfeeding mothers otherwise to the children) was associated with a lower weight-gain particularly in 4-year-old children (13). Nevertheless, not all investigators have detected an association between probiotics supplementation during pregnancy and child’s weight, as indicated in a recent meta-analysis (16), although in these trials, the growth outcome was evaluated via birthweight. Contrary to our hypothesis, we did not detect an association between the probiotic supplementation and child’s body fat percentage. Our result is partly in line with 1 study (New Zealand, n = 230) (32) demonstrating no difference in body fat percentage (measured by the Pea Pod-system) of children at birth as a response to probiotics supplementation (minimum 6.5 × 10^9^ colony-forming units of *L rhamnosus* GG and *B lactis* BB-12) to obese pregnant women. It is noteworthy that in our study the number of children (n = 73), whose body composition was measured, was relatively low which affects the trial’s power to detect differences between the groups. Additionally, the probiotic’s impacts can be different depending on the bacteria strain used. Taken together, there is thus far limited evidence available on the association of the probiotics consumption during pregnancy and child’s body composition. Considering the health burden attributable to obesity, this is a topic that deserves further investigation.

The presence of obesity has been associated with low-grade inflammation in the body (33), thus the beneficial effects of probiotics on child’s weight may be due to their anti-inflammatory effects during pregnancy (21,34). The inflammatory markers can cross the placenta and affect the development of metabolic pathways in the fetus that could later lead to the development of various diseases, such as obesity (35). Probiotics may also influence child’s weight development by beneficially modifying the composition of intestinal microbiota (36,37). That leads to production of short-chain fatty acids which can influence the metabolic programming of the fetus (38). Additionally, probiotics are able to decrease DNA methylation of obesity- and weight gain-related genes in both mothers and children that could lead to silencing of these genes and to a lower weight in the children (39).

As far as we are aware, this is the first study investigating the combined effects of probiotics and fish oil on child’s growth, as no previous literature is available. We observed that the children whose mothers received the combination of probiotics and fish oil were shorter than those of mothers who received placebo over the 24-months’ study period. It is of note that the mean growth was within the normal reference range, thus the result may be interpreted to reflect potentially an adverse effect of the combination of these supplements. However, rapid growth in childhood is not necessarily desirable, as it may associate with overweight in later life (40). Indeed, previous evidence indicates that there is a positive association between overweight and height in infancy (41,42). Here, children of mothers in the combined probiotics and fish oil-group were less often overweight when compared to those in the placebo-group. Furthermore, it should be remembered that the child’s growth is a dynamic process affected by environmental but also genetic factors especially during the first years of his/her life (43).

We did not find any evidence that fish oil alone affected children’s overweight odds or body fat percentage. The reason for our finding is not clear, but one explanation could be that all the women were overweight/obese, which could have attenuated the effect of n-3 fatty acids. This has been previously revealed in 1 study, where LC-PUFA (DHA 0.80 g, EPA 1.20 g/day) supplementation during pregnancy led to lower plasma concentration of LC-PUFA in women with obesity compared to lean women (44). Similarly, some previous investigators have not detected any association between fish oil supplementation, using lower doses (DHA = 0.80 g/day, EPA = 0.10 g/day; DHA = 0.40 g/day; DHA = 0.8 g/day, EPA = 1.2 g/day) than provided in our study (DHA = 1.9 g, EPA = 0.22 g/day), and weight, fat percentage or mass, height and head circumference at birth (8,15), and adiposity in 3- or 5-year-old children (14). In contrast, others have shown that DHA supplementation, even at a low dose (200 mg/day from 21st gestational weeks until 3 months postpartum) and a higher LC-PUFA intake from the diet during pregnancy is associated with a lower weight, BMI, and obesity risk in 21- and 36-month-old children (9,45). It is of note that the amount of EPA and DHA, the intervention duration, and the targeted intervention population as well as the child’s age could account for these apparently discrepant findings.

Our study has various strengths. We used data from a randomized, placebo-controlled, double-blind clinical trial that enabled us to take into consideration possible confounding factors in the statistical analysis, although we did not adjust for breast-feeding, due to partially missing data, which may contribute to child’s growth (46). We also had detailed longitudinal data available on the children’s height, weight, and head circumference up to 24 months. We measured the children’s body composition with a sensitive air displacement plethysmography, comparable to underwater-weighing, the state-of-the-art method for body composition analysis. As overweight and obesity are global public health problems (47), our study provides essential knowledge about whether supplementation of fish oil, probiotics, and for the first time their combination in pregnant women with those metabolic disturbances could regulate the child’s growth, especially the overweight risk. Our study also has limitations. All study women had overweight/obesity, and maternal BMI is known to associate with children’s adiposity. However, our study sample represents the all-too-common clients arriving in Finnish maternal clinics nowadays (48). The women included in the study had higher education level and they smoked less likely before pregnancy when compared to those not included (Table 3, Supplemental Digital Content 4, http://links.lww.com/MPG/C998). Highly educated women may have an overall healthier lifestyle, including diet that could also itself reflect child feeding practices and thus child growth. However, the percentage of girls with overweight in our study (17.1%) is consistent to that of Finnish population (18%) (49), although the percentage among boys is lower (19.7% vs 29%). Furthermore, limitations were that the body fat percentage was available in only one-third of the children, and we collected the growth data from clinic cards. Although predefined in the trial, the outcomes assessed here were secondary outcomes, and thus no power-calculation could be performed.

## CONCLUSIONS

We conclude that the probiotics consumption on their own or in combination with fish oil from early pregnancy onwards could be beneficial for lowering the overweight odds of 24-month-old children born to mothers with overweight/obesity. Probiotics and fish oil administration together seemed to lead slower growth of children, although within the normal reference range. The clinical significance of this finding needs further elucidation but may support our finding that these children less likely became overweight when they are 24 months old. Our findings could be utilized in the dietary counseling of the most vulnerable women, that is, those with overweight/obesity, as it is putative that this population would mostly benefit from the intervention (19,21,50).

## Acknowledgments

We thank families who participated in the FOPP study, and Ewen MacDonald for the English language revision.

## Supplementary Material

## References

[1] Parrettini S Caroli A Torlone E. Nutrition and metabolic adaptations in physiological and complicated pregnancy: focus on obesity and gestational diabetes. Front Endocrinol. 2020;11:611929. doi:10.3389/FENDO.2020.611929. 10.3389/fendo.2020.611929 PMC7793966 33424775

[2] Catalano PM Shankar K. Obesity and pregnancy: mechanisms of short term and long term adverse consequences for mother and child. BMJ. 2017;356:1. doi:10.1136/BMJ.J1. 10.1136/bmj.j1 PMC6888512 28179267

[3] Catalano PM McIntyre HD Cruickshank JK. The hyperglycemia and adverse pregnancy outcome study: associations of GDM and obesity with pregnancy outcomes. Diabetes Care. 2012;35:780–6. doi:10.2337/DC11-1790. 22357187 10.2337/dc11-1790 PMC3308300

[4] Godfrey KM Reynolds RM Prescott SL. Influence of maternal obesity on the long-term health of offspring. Lancet Diabetes Endocrinol. 2017;5:53–64. doi:10.1016/S2213-8587(16)30107-3. 27743978 10.1016/S2213-8587(16)30107-3 PMC5245733

[5] de Boo HA Harding JE. The developmental origins of adult disease (Barker) hypothesis. Aust N Z J Obstet Gynaecol. 2006;46:4–14. doi:10.1111/j.1479-828x.2006.00506.x. 16441686 10.1111/j.1479-828X.2006.00506.x

[6] Carlson SE Colombo J Gajewski BJ. DHA supplementation and pregnancy outcomes. Am J Clin Nutr. 2013;97:808–15. doi:10.3945/AJCN.112.050021. 23426033 10.3945/ajcn.112.050021 PMC3607655

[7] Vinding RK Stokholm J Sevelsted A. Fish oil supplementation in pregnancy increases gestational age, size for gestational age, and birth weight in infants: a randomized controlled trial. J Nutr. 2019;149:628–34. doi:10.1093/JN/NXY204. 30418579 10.1093/jn/nxy204

[8] Monthé-Drèze C Sen S de Mouzon SH. Effect of Omega-3 supplementation in pregnant women with obesity on newborn body composition, growth and length of gestation: a randomized controlled pilot study. Nutrients. 2021;13:1–19. doi:10.3390/NU13020578. 10.3390/nu13020578 PMC7916127 33572368

[9] Donahue SMA Rifas-Shiman SL Gold DR. Prenatal fatty acid status and child adiposity at age 3 y: results from a US pregnancy cohort. Am J Clin Nutr. 2011;93:780–8. doi:10.3945/AJCN.110.005801. 21310834 10.3945/ajcn.110.005801 PMC3057547

[10] Michael DR Jack AA Masetti G. A randomised controlled study shows supplementation of overweight and obese adults with lactobacilli and bifidobacteria reduces bodyweight and improves well-being. Sci Rep. 2020;10:4183. doi:10.1038/s41598-020-60991-7. 32144319 10.1038/s41598-020-60991-7 PMC7060206

[11] Michael DR Davies TS Jack AA. Daily supplementation with the Lab4P probiotic consortium induces significant weight loss in overweight adults. Sci Rep. 2021;11:5. doi:10.1038/s41598-020-78285-3. 33408364 10.1038/s41598-020-78285-3 PMC7788077

[12] Sudha MR Ahire JJ Jayanthi N. Effect of multi-strain probiotic (UB0316) in weight management in overweight/obese adults: a 12-week double blind, randomised, placebo-controlled study. Benef Microbes. 2019;10:855–66. doi:10.3920/BM2019.0052. 31965834 10.3920/BM2019.0052

[13] Luoto R Kalliomäki M Laitinen K Isolauri E. The impact of perinatal probiotic intervention on the development of overweight and obesity: follow-up study from birth to 10 years. Int J Obes. 2010;34:1531–7. doi:10.1038/IJO.2010.50. 10.1038/ijo.2010.50 20231842

[14] Muhlhausler BS Yelland LN McDermott R. DHA supplementation during pregnancy does not reduce BMI or body fat mass in children: follow-up of the DHA to Optimize Mother Infant Outcome randomized controlled trial. Am J Clin Nutr. 2016;103:1489–96. doi:10.3945/AJCN.115.126714. 27030533 10.3945/ajcn.115.126714

[15] Khandelwal S Kondal D Chaudhry M. Prenatal maternal docosahexaenoic acid (DHA) supplementation and newborn anthropometry in India: findings from DHANI. Nutrients. 2021;13:7301–12. doi:10.3390/NU13030730. 10.3390/nu13030730 PMC7996222 33668849

[16] Pérez-Castillo IM Fernández-Castillo R Lasserrot-Cuadrado A. Reporting of perinatal outcomes in probiotic randomized controlled trials. a systematic review and meta-analysis. Nutrients. 2021;13:2561–24. doi:10.3390/NU13010256. 33477352 10.3390/nu13010256 PMC7830438

[17] Mokkala K Vahlberg T Houttu N Koivuniemi E Lahti L Laitinen K. Impact of combined consumption of fish oil and probiotics on the serum metabolome in pregnant women with overweight or obesity. EBioMedicine. 2021;73:103655. doi:10.1016/J.EBIOM.2021.103655. 34740110 10.1016/j.ebiom.2021.103655 PMC8577343

[18] Lalia AZ Lanza IR. Insulin-sensitizing effects of omega-3 fatty acids: lost in translation? Nutrients. 2016;8:329. doi:10.3390/NU8060329. 27258299 10.3390/nu8060329 PMC4924170

[19] Haghiac M Yang XH Presley L. Dietary omega-3 fatty acid supplementation reduces inflammation in obese pregnant women: A randomized double-blind controlled clinical trial. PLoS One. 2015;10:e0137309. doi:10.1371/journal.pone.0137309. 26340264 10.1371/journal.pone.0137309 PMC4560373

[20] Pan YQ Zheng QX Jiang XM. Probiotic supplements improve blood glucose and insulin resistance/sensitivity among healthy and GDM pregnant women: a systematic review and meta-analysis of randomized controlled trials. Evid Based Complement Alternat Med. 2021;2021. doi:10.1155/2021/9830200. 10.1155/2021/9830200 PMC8481047 34603479

[21] Zheng HJ Guo J Jia Q. The effect of probiotic and synbiotic supplementation on biomarkers of inflammation and oxidative stress in diabetic patients: a systematic review and meta-analysis of randomized controlled trials. Pharmacol Res. 2019;142:303–13. doi:10.1016/J.PHRS.2019.02.016. 30794924 10.1016/j.phrs.2019.02.016

[22] Pellonperä O Mokkala K Houttu N. Efficacy of fish oil and/or probiotic intervention on the incidence of gestational diabetes mellitus in an at-risk group of overweight and obese women: a randomized, placebo-controlled, double-blind clinical trial. Diabetes Care. 2019;42:1009–17. doi:10.2337/DC18-2591. 30967436 10.2337/dc18-2591

[23] Pajunen L Korkalo L Koivuniemi E. A healthy dietary pattern with a low inflammatory potential reduces the risk of gestational diabetes mellitus. Eur J Nutr. 2022;61:1477–90. doi:10.1007/S00394-021-02749-Z. 34846602 10.1007/s00394-021-02749-z PMC8921111

[24] Dekker J Collett M Prasad J Gopal P. Functionality of probiotics – potential for product development. Forum Nutr. 2007;60:196–208. doi:10.1159/000107196. 17684416 10.1159/000107196

[25] Amar J Chabo C Waget A. Intestinal mucosal adherence and translocation of commensal bacteria at the early onset of type 2 diabetes: molecular mechanisms and probiotic treatment. EMBO Mol Med. 2011;3:559–72. doi:10.1002/EMMM.201100159. 21735552 10.1002/emmm.201100159 PMC3265717

[26] Klein A Friedrich U Vogelsang H Jahreis G. *Lactobacillus acidophilus* 74-2 and *Bifidobacterium animalis* subsp *lactis* DGCC 420 modulate unspecific cellular immune response in healthy adults. Eur J Clin Nutr. 2008;62:584–93. doi:10.1038/SJ.EJCN.1602761. 17440520 10.1038/sj.ejcn.1602761

[27] Larqué E Gil-Sánchez A Prieto-Sánchez MT. Omega 3 fatty acids, gestation and pregnancy outcomes. Br J Nutr. 2012;107:S77–84. 22591905 10.1017/S0007114512001481

[28] Karvonen M Hannila ML Saari A Dunkel L. New Finnish reference for head circumference from birth to 7 years. Ann Med. 2012;44:369–74. doi:10.3109/07853890.2011.558519. 21495784 10.3109/07853890.2011.558519

[29] Saari A Sankilampi U Hannila ML Kiviniemi V Kesseli K Dunkel L. New Finnish growth references for children and adolescents aged 0 to 20 years: length/height-for-age, weight-for-length/height, and body mass index-for-age. Ann Med. 2011;43:235–48. doi:10.3109/07853890.2010.515603. 20854213 10.3109/07853890.2010.515603

[30] Sankilampi U Hannila ML Saari A Gissler M Dunkel L. New population-based references for birth weight, length, and head circumference in singletons and twins from 23 to 43 gestation weeks. Ann Med. 2013;45:446–54. doi:10.3109/07853890.2013.803739. 23768051 10.3109/07853890.2013.803739

[31] Fomon SJ Haschke F Ziegler EE. Body composition of reference children from birth to age 10 years. Am J Clin Nutr. 1982;35:1169–75. doi:10.1093/AJCN/35.5.1169. 7081099 10.1093/ajcn/35.5.1169

[32] Okesene-Gafa KAM Li M McKinlay CJD. Effect of antenatal dietary interventions in maternal obesity on pregnancy weight-gain and birthweight: Healthy Mums and Babies (HUMBA) randomized trial. Am J Obstet Gynecol. 2019;221:152.e1–152.e13. doi:10.1016/J.AJOG.2019.03.003. 10.1016/j.ajog.2019.03.003 30878323

[33] Pantham P Aye ILMH Powell TL. Inflammation in maternal obesity and gestational diabetes mellitus. Placenta. 2015;36:709–15. doi:10.1016/J.PLACENTA.2015.04.006. 25972077 10.1016/j.placenta.2015.04.006 PMC4466145

[34] Zhou L Ding C Wu J. Probiotics and synbiotics show clinical efficacy in treating gestational diabetes mellitus: a meta-analysis. Prim Care Diabetes. 2021;15:937–47. doi:10.1016/J.PCD.2021.08.005. 34417122 10.1016/j.pcd.2021.08.005

[35] Parisi F Milazzo R Savasi VM. Maternal low-grade chronic inflammation and intrauterine programming of health and disease. Int J Mol Sci. 2021;22:1–16. doi:10.3390/IJMS22041732. 10.3390/ijms22041732 PMC7914818 33572203

[36] Wiciński M Gębalski J Gołębiewski J. Probiotics for the treatment of overweight and obesity in humans—a review of clinical trials. Microorganisms. 2020;8:11481–26. doi:10.3390/MICROORGANISMS8081148. 10.3390/microorganisms8081148 PMC7465252 32751306

[37] Fu Y Wang Y Gao H. Associations among dietary omega-3 polyunsaturated fatty acids, the gut microbiota, and intestinal immunity. Mediators Inflamm. 2021;2021:8879227. 33488295 10.1155/2021/8879227 PMC7801035

[38] Ziętek M Celewicz Z Szczuko M. Short-chain fatty acids, maternal microbiota and metabolism in pregnancy. Nutrients. 2021;13:1244. doi:10.3390/NU13041244. 33918804 10.3390/nu13041244 PMC8069164

[39] Vähämiko S Laiho A Lund R Isolauri E Salminen S Laitinen K. The impact of probiotic supplementation during pregnancy on DNA methylation of obesity-related genes in mothers and their children. Eur J Nutr. 2019;58:367–77. doi:10.1007/S00394-017-1601-1. 29299736 10.1007/s00394-017-1601-1

[40] Stovitz SD Demerath EW Hannan PJ Lytle LA Himes JH. Growing into obesity: patterns of height growth in those who become normal weight, overweight or obese as young adults. Am J Hum Biol. 2011;23:635–41. doi:10.1002/AJHB.21191. 21630370 10.1002/ajhb.21191 PMC3152584

[41] Papadimitriou A Gousi T Giannouli O Nicolaidou P. The growth of children in relation to the timing of obesity development. Obesity (Silver Spring). 2006;14:2173–6. doi:10.1038/OBY.2006.254. 17189543 10.1038/oby.2006.254

[42] Holmgren A Niklasson A Nierop AFM. Pubertal height gain is inversely related to peak BMI in childhood. Pediatr Res. 2017;81:448–54. doi:10.1038/PR.2016.253. 27861464 10.1038/pr.2016.253

[43] Jelenkovic A Sund R Hur YM. Genetic and environmental influences on height from infancy to early adulthood: an individual-based pooled analysis of 45 twin cohorts. Sci Rep. 2016;6:28496. doi:10.1038/SREP28496. 27333805 10.1038/srep28496 PMC4917845

[44] Monthé-Drèze C Penfield-Cyr A Smid MC Sen S. Maternal pre-pregnancy obesity attenuates response to omega-3 fatty acids supplementation during pregnancy. Nutrients. 2018;10:1908. doi:10.3390/NU10121908. 30518052 10.3390/nu10121908 PMC6315963

[45] Bergmann R Bergmann KE Haschke-Becher E. Does maternal docosahexaenoic acid supplementation during pregnancy and lactation lower BMI in late infancy? J Perinat Med. 2007;35:295–300. 17547539 10.1515/JPM.2007.085

[46] Durmuş B van Rossem L Duijts L. Breast-feeding and growth in children until the age of 3 years: the Generation R Study. Br J Nutr. 2011;105:1704–11. doi:10.1017/S0007114510005374. 21276279 10.1017/S0007114510005374

[47] di Cesare M Bentham J Stevens GA. Trends in adult body-mass index in 200 countries from 1975 to 2014: a pooled analysis of 1698 population-based measurement studies with 19.2 million participants. Lancet. 2016;387:1377–96. doi:10.1016/S0140-6736(16)30054-X. 27115820 10.1016/S0140-6736(16)30054-X PMC7615134

[48] Finnish Institute for Health and Welfare. Perinatal Statistics – Parturients, Deliveries and Newborns 2020. Statistical Report 48/2020. Official Statistics of Finland, Perinatal Statistics. Finland, Finnish Institute for Health and Welfare; 2020.

[49] Finnish Institute for Health and Welfare. Child and Adolescent Overweight and Obesity 2020 (available only in Finnish and Swedish). Statistical report 37/2021. Finland, Finnish Institute for Health and Welfare; 2021.

[50] Pot GK Brouwer IA Enneman A. No effect of fish oil supplementation on serum inflammatory markers and their interrelationships: a randomized controlled trial in healthy, middle-aged individuals. Eur J Nutr. 2009;63:1353–9. doi:10.1038/ejcn.2009.63. 10.1038/ejcn.2009.63 19623203

