# Supplementary material for: Fish Oil And/Or Probiotics Intervention in Overweight/Obese Pregnant Women and Overweight Risk in 24-Month-Old Children
Source: J Pediatr Gastroenterol Nutr. 2022 Nov 23;76(2):218–26. doi: 10.1097/MPG.0000000000003659 (PMC9848211; doi:10.1097/MPG.0000000000003659)
Supplement: Supplementary file 1 [file mpg-76-218-s001.pdf]

Supplemental Digital Content 1

Fish Oil And/Or Probiotics Intervention in Overweight/Obese Pregnant Women and Overweight Risk in 24-Month-Old Children

Journal of Pediatric Gastroenterology and Nutrition

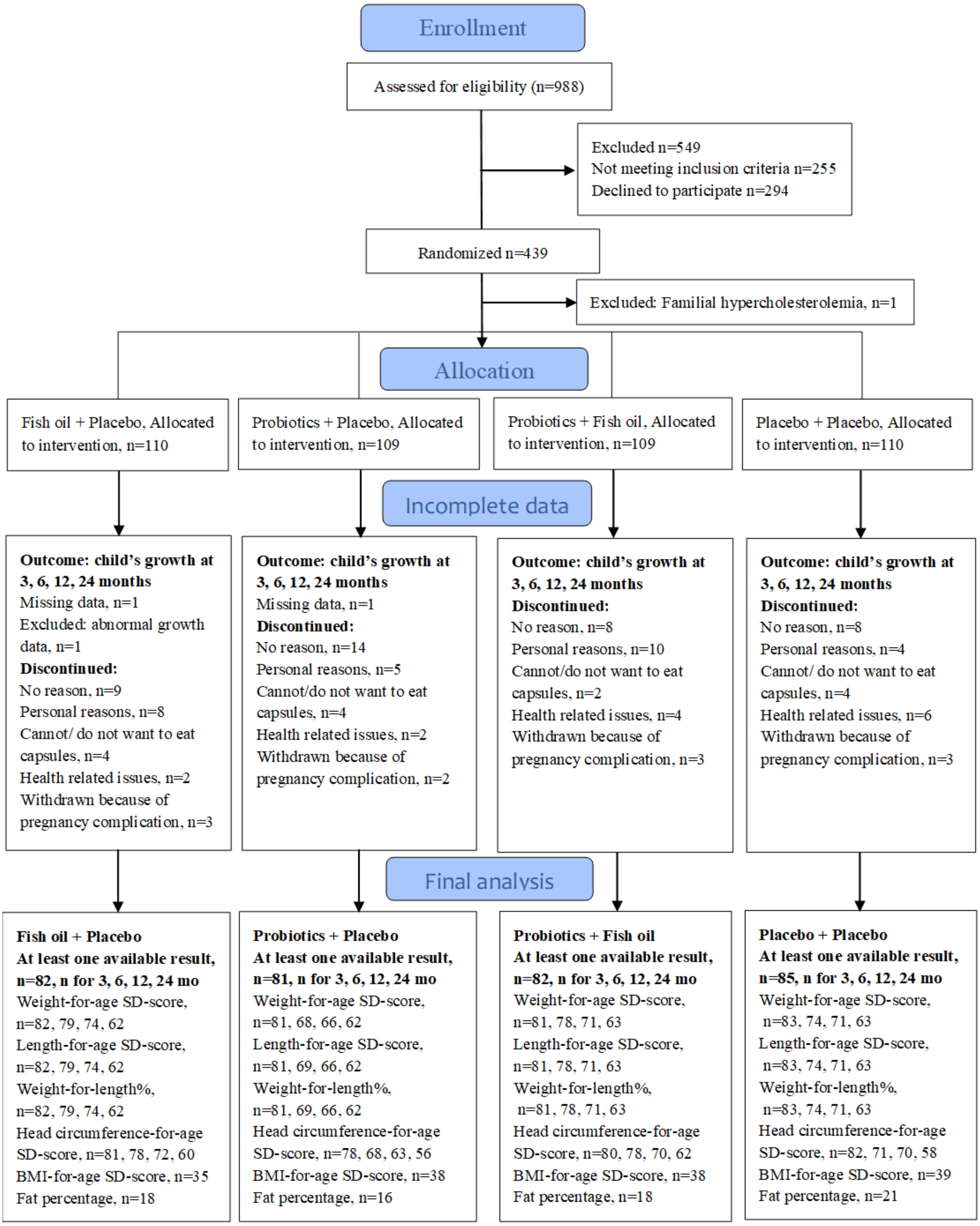

Figure, Flow diagram of the present study
