# Supplementary material for: Fish Oil And/Or Probiotics Intervention in Overweight/Obese Pregnant Women and Overweight Risk in 24-Month-Old Children
Source: J Pediatr Gastroenterol Nutr. 2022 Nov 23;76(2):218–26. doi: 10.1097/MPG.0000000000003659 (PMC9848211; doi:10.1097/MPG.0000000000003659)
Supplement: Supplementary file 2 [file mpg-76-218-s002.pdf]

Supplemental Digital Content 2

Fish Oil And/Or Probiotics Intervention in Overweight/Obese Pregnant Women and Overweight Risk in24-Month-Old Children

Journal of Pediatric Gastroenterology and Nutrition

Table, Association between maternal groups receiving fish oil vs. non-fish oil or probiotics vs. non-probiotics with children's overweight status at 24 months

Growth measures	Fish oil effect		Probiotics effect		Fish oil × Probiotics interaction effect
		Adjusted p †		Adjusted p †	Adjusted p †
**Weight-for-height%**	n (%)		n (%)		
Normal+Underweight	104 (83.2)		108 (86.4)		
Overweight+Obese	21 (16.8)		17 (13.6)		
Adjusted OR (95% CI)	0.96 (0.49–1.90)	0.917	0.48 (0.25–0.95)	0.035	0.397
**Weight-for-age SD-score**					
Normal+Underweight	111 (88.8)		111 (88.8)		
Overweight+Obese	14 (11.2)		14 (11.2)		
Adjusted OR (95% CI)	0.56 (0.27–1.18)	0.125	0.42 (0.20–0.88)	0.021	0.701
**BMI-for-age SD-score**					
Normal+Underweight	54 (75.0)		59 (78.8)		
Overweight+Obese	18 (25.0)		16 (21.3)		
Adjusted OR (95% CI)	1.18 (0.54–2.58)	0.680	0.67 (0.31–1.46)	0.312	0.130

Data are presented as adjusted OR (95% CI).

† Binary logistic regression model for overweight with non-fish oil or non-probiotics as the reference categories. Adjusted for maternal smoking status before pregnancy, child's birth weight, and child's age at the measurement (weight-for-height%).

BMI, body mass index; CI, confidence interval; OR, odds ratio; SD-score, standard deviation score.

Fish oil (fish oil+placebo and probiotics+fish oil), non-fish oil (probiotics+placebo and placebo+placebo).

Probiotics (probiotics+placebo and fish oil+probiotics), non-probiotics (placebo+fish oil and placebo+placebo).
