# Supplementary material for: Fish Oil And/Or Probiotics Intervention in Overweight/Obese Pregnant Women and Overweight Risk in 24-Month-Old Children
Source: J Pediatr Gastroenterol Nutr. 2022 Nov 23;76(2):218–26. doi: 10.1097/MPG.0000000000003659 (PMC9848211; doi:10.1097/MPG.0000000000003659)
Supplement: Supplementary file 3 [file mpg-76-218-s003.pdf]

Supplemental Digital Content 3

Fish Oil And/Or Probiotics Intervention in Overweight/Obese Pregnant Women and Overweight Risk in 24-Month-Old Children

Journal of Pediatric Gastroenterology and Nutrition

Table, Association between children's growth markers and maternal groups receiving fish oil vs. non-fish oil and probiotics vs. non-probiotics

Growth measures		Fish oil	Non-fish oil	Fish oil effect			Probiotics	Non-probiotics	Probiotics effect		Fish oil × Probiotics interaction effect
	n	Adjusted mean (SE) or geometric mean (95% CI)	Adjusted mean (SE) or geometric mean (95% CI)	Adjusted mean difference or proportional difference (95% CI)	Adjusted p †	n	Adjusted mean (SE) or geometric mean (95% CI)	Adjusted mean (SE) or geometric mean (95% CI)	Adjusted mean difference or proportional difference (95% CI)	Adjusted p †	Adjusted p †
**3 months**											
Height SD-score	163/163	-0.24 (0.09)	-0.18 (0.09)	-0.02 (-0.23–0.19)	0.834	161/165	-0.18 (0.09)	-0.20 (0.09)	0.02 (-0.18–0.23)	0.843	0.155
Weight-for-height%	163/163	4.03 (0.84)	3.32 (0.73)	0.72 (-1.17–2.61)	0.456	161/165	3.42 (0.79)	3.93 (0.77)	-0.50 (-2.34–1.34)	0.590	0.758
Weight-for-age SD-score	163/163	0.04 (0.09)	0.01 (0.08)	0.03 (-0.16–0.23)	0.739	161/165	-0.01 (0.08)	0.06 (0.08)	-0.05 (-0.25–0.14)	0.578	0.341
Head circumference-for-age SD-score	161/159	-0.001 (0.10)	-0.02 (0.09)	-0.02 (-0.22–0.25)	0.890	157/163	-0.05 (0.10)	-0.02 (0.09)	0.03 (-0.20–0.26)	0.811	0.981
**6 months**											
Height SD-score	157/143	-0.16 (0.10)	-0.19 (0.10)	0.03 (-0.21–0.26)	0.832	147/153	-0.21 (0.10)	-0.14 (0.10)	-0.08 (-0.31–0.15)	0.498	0.791
Weight-for-height%	157/143	4.95 (0.88)	4.80 (0.79)	0.15 (-1.84–2.15)	0.882	147/153	4.42 (0.84)	5.32 (0.82)	-0.90 (-2.84–1.05)	0.366	0.779
Weight-for-age SD-score	157/142	0.19 (0.09)	0.13 (0.08)	0.05 (-0.16–0.26)	0.615	146/153	0.10 (0.09)	0.22 (0.09)	-0.13 (-0.33–0.08)	0.228	0.701
Head circumference-for-age SD-score	156/139	-0.02 (0.11)	0.08 (0.10)	-0.06 (-0.30–0.18)	0.618	146/149	-0.01 (0.10)	0.09 (0.10)	-0.09 (-0.33–0.14)	0.437	0.393
**12 months**											

Height SD-score	145/ 137	0.10 (0.11)	-0.05 (0.10)	-0.05 (-0.29–0.19)	0.681	137/ 145	-0.18 (0.10)	0.02 (0.10)	-0.20 (-0.44–0.03)	0.092	0.297
Weight-for-height%	145/ 137	3.98 (0.87)	4.57 (0.77)	-0.60 (-2.55–1.35)	0.543	137/ 145	3.92 (0.82)	4.63 (0.81)	-0.70 (-2.61–1.20)	0.467	0.856
Weight-for-age SD-score	145/ 137	0.17 (0.10)	0.23 (0.09)	-0.07 (-0.29–0.16)	0.561	137/ 145	0.11 (0.09)	0.29 (0.09)	-0.18 (-0.39–0.04)	0.105	0.601
Head circumference-for-age SD-score	142/ 133	-0.13 (0.11)	0.10 (0.10)	-0.23 (-0.48–0.03)	0.084	133/ 142	-0.07 (0.11)	0.04 (0.11)	-0.11 (-0.36–0.14)	0.407	0.703
**24 months**											
Height SD-score	125/ 125	-0.21 (0.11)	0.03 (0.10)	-0.24 (-0.50–0.02)	0.066	125/ 125	-0.19 (0.11)	0.001 (0.11)	-0.19 (-0.44–0.07)	0.147	0.515
Weight-for-height%	125/ 125	4.85 (0.92)	3.40 (0.80)	1.45 (-0.65–3.55)	0.175	125/ 125	2.93 (0.86)	5.32 (0.86)	-2.39 (-4.44–(-0.34))	0.023	0.629
Weight-for-age SD-score	125/ 125	0.19 (0.11)	0.21 (0.10)	-0.02 (-0.26–0.21)	0.840	125/ 125	0.05 (0.10)	0.34 (0.10)	-0.29 (-0.52–(-0.06))	0.015	0.957
Head circumference-for-age SD-score	122/ 114	-0.15 (0.12)	0.11 (0.10)	-0.26 (-0.53–0.01)	0.061	118/ 118	-0.07 (0.11)	0.03 (0.11)	-0.09 (-0.36–0.17)	0.494	0.695
BMI-for-age SD-score	72/ 77	0.45 (0.15)	0.38 (0.13)	0.07 (-0.27–0.41)	0.700	75/ 74	0.27 (0.14)	0.55 (0.14)	-0.28 (-0.62–0.06)	0.108	0.688
Fat percentage[1]	36/ 37	22.4 (19.4; 25.8)	22.4 (19.5; 25.8)	1.00 (0.84–1.20)	0.987	34/ 39	22.3 (19.2; 25.8)	22.6 (19.7; 25.8)	0.99 (0.82–1.19)	0.875	0.226

Data are presented as adjusted mean (SE), adjusted mean difference (95% CI) or [1] adjusted geometric mean (95% CI), [1] proportional difference for adjusted geometric mean (95% CI). Fat percentage ln transformed for the analysis due to skewed distribution.

[†] General linear model. Adjusted for maternal smoking status before pregnancy, child's birth weight, and child's age at the measurement (weight-for-height%).

BMI, body mass index; CI, confidence interval; SD-score, standard deviation score; SE, standard error.

Fish oil (fish oil+placebo and fish oil+probiotics), non-fish oil (probiotics+placebo and placebo+placebo).

Probiotics (probiotics+placebo and probiotics+fish oil), non-probiotics (placebo+fish oil and placebo+placebo).
