# Supplementary material for: Fish Oil And/Or Probiotics Intervention in Overweight/Obese Pregnant Women and Overweight Risk in 24-Month-Old Children
Source: J Pediatr Gastroenterol Nutr. 2022 Nov 23;76(2):218–26. doi: 10.1097/MPG.0000000000003659 (PMC9848211; doi:10.1097/MPG.0000000000003659)
Supplement: Supplementary file 4 [file mpg-76-218-s004.pdf]

Supplemental Digital Content 4

Fish Oil And/Or Probiotics Intervention in Overweight/Obese Pregnant Women and Overweight Risk in 24-Month-Old Children

Journal of Pediatric Gastroenterology and Nutrition

Table, Comparable baseline characteristics between the mothers whose children were included in the study and the mothers whose children were not included.

Characteristics	n	Included	Not included	p
Age (yrs) [1]	330/107	30.7±4.5	30.1±4.9	0.212
College or university education [2]	328/62	211 (64.3)	27 (43.5)	0.003
Primiparity [2]	330/107	161 (48.8)	48 (44.9)	0.505
Smoked before pregnancy [2]	329/63	57 (17.3)	29 (46.0)	<0.001
Pre-pregnancy BMI (kg/m^2) [3]	330/107	28.7 (26.5; 31.8)	28.5 (26.2; 32.6)	0.794
Overweight [2]<br>Obese [2]	330/107	200 (60.6)<br>130 (39.4)	66 (61.7)<br>41 (38.3)	0.909
Gestational weeks [1]	330/107	13.9±2.1	13.6±2.3	0.262

Data are presented as [1] mean ± SD, [2] frequency (%) or [3] median (interquartile range).
[†] Inpedendent samples t-Test for normally distributed variables, otherwise Mann-Whitney U-test. Fisher exact test for categorical variables.

BMI, body mass index; GDM, gestational diabetes mellitus; LGA, large for gestational age; SGA, small for gestational age; SD, standard deviation
